# Exposure to Toenail Heavy Metals and Child Behavior Problems in Nine-Year-Old Children: A Cross-Sectional Study

**DOI:** 10.3390/ijerph17114120

**Published:** 2020-06-09

**Authors:** Shamshad Karatela, Christin Coomarasamy, Janis Paterson, Neil I. Ward

**Affiliations:** 1 Faculty of Medicine, University of Queensland, Herston, QLD 4006, Australia; 2 Ko awatea, Counties Manukau Health, Private Bag 93311, Auckland 1640, New Zealand; Christin.coomarasamy@middlemore.co.nz; 3 School of Public Health and Psychosocial Studies, Auckland University of Technology, Auckland 0627, New Zealand; janis.paterson@aut.ac.nz; 4 Department of Chemistry FEPS, University of Surrey, Guildford, Surrey GU2 7XH, UK; n.ward@surrey.ac.uk

**Keywords:** behavior problems, nail biomarker, heavy metals

## Abstract

Behavioral problems are multifactorial and includes perinatal, maternal, family, parenting, socio-economic and personal risk factors, but less is known about the association of postnatal heavy metals on children’s behavioral problems in Pacific Island children. Methods: A cohort of eligible nine-year-old children within a Pacific Island Families longitudinal study were recruited for a cross-sectional study. Child behavior problems were assessed using the child behavior checklist. Heavy metals (including Ni, Cu, Pb, Al, Cr and Cd) were determined in toenails, after acid digestion and analyzed using inductively coupled plasma mass spectrometry. Other factors such as lifestyle (smoking in pregnancy), health outcomes (obesity, health status), demographics (gender, ethnicity, parents’ marital status) and socioeconomic status (household income levels) were also collected. The statistical analysis included *t*-tests for independent sample and Mann–Whitney U-test, and chi-square or Fisher’s exact tests of independence for comparisons of the proportions. Regression models tested the hypothesized risk factors for behavior outcomes. Results: This observational study enrolled 278 eligible Pacific Island children living in Auckland, New Zealand. The prevalence of behavioral problems in the clinical range was high (22%) but there was no significant association between heavy metals in toenails and adverse behavioral outcomes. Conclusion: Regular monitoring and assessments of children for environmental risk factors, as well as social and lifestyle factors for behavior problems, continues. Alternative indicators of exposure to heavy metal should be evaluated.

## 1. Introduction

Child behavior problems, a developmental disorder, are a cause of lifelong health morbidity with negative consequences for adult as well as child health. Around 5–10% of children around the world are affected by developmental disorders [1]. Behavioral problems in children can be complex as they involve a range of other factors such as biological, psychosocial and environmental factors [2,3,4]. However, evidence is increasing that adverse environmental exposures have a substantial role in the initiation and progression of childhood behavior problems [5]. Heavy metals occur naturally; however, with climate change heavy metal contamination is getting worse [6]. Many of these chemicals can bio-accumulate in predators, and eventually reach humans who may consume such products like fish. Third-hand smoking, another source of heavy metal exposure, can remain in places where smoking may have occurred for very long periods. Children are more vulnerable due to their small size and their hand-to-mouth habits which put them at risk of exposure to these contaminants. In 2001, a survey estimated that 0.9% of Pacific children between the ages of 0 to 14 years had developmental disabilities, particularly an intellectual disability [7]. Another survey conducted between 2005 and 2009 found that the number of Pacific children and young people with developmental delays was higher than for their Māori, European and Asian counterparts [8]. A survey conducted between 2005 and 2009 found that mental health issues are experienced by Pacific people at a higher rate than among the general population, especially in Pacific people who were born in New Zealand (NZ), 37% of whom have mental health disorders compared to 15% of those who migrated from Pacific Island countries [9].

Many studies have reported an association between heavy metal exposures and behavioral problems. Specifically, research in neurotoxin (such as lead (Pb)) has demonstrated the effect that toxins can have on the absorption of various essential elements, thus reducing the bioavailability of essential elements, which can potentially lead to behavioral problems [10]. Cadmium (Cd) is known to cause toxicity to humans, especially children. Studies have shown that at higher levels, there is an increased risk of learning difficulties and special education [11] as well as aggressive behavior [12]. Its adverse effects during pregnancy in some studies have been documented [13,14] while others have shown no effects [15]. Other elements such as nickel (Ni) and chromium (Cr) at minute levels are considered essential; however, certain forms of these metals can be toxic and carcinogenic [16]. The associations between postnatal Ni, Cr exposures and child behavior problems have not been documented before. However, Cr is known to cause respiratory effects [17], dermatological effects [18], and has also been linked to cancer [19]. Ni is known to cause certain kinds of cancers [20]. However, with regards to behavior problems, a systematic review showed that a weak association remained between Ni and autism, but no associations were observed in child behavioral outcomes [16]. Aluminum is non-essential to humans; however, exposure to this metal is very common especially via food, medications or cooking utensils. Aluminum is blocked by the blood–brain barrier; however, preterm brains are more susceptible as they are still developing. A study has shown that preterm infants exposed to Al via feeding on intravenous feeding solution resulted in impaired neurological development [21]. Studies have shown that the long-term effects of prolonged aluminum exposure lead to Alzheimer’s disease [22]. Copper (Cu) is an essential element at minute levels, but at high levels and longer exposure duration it can have deleterious effects, especially on children. It is known to cause gastrointestinal disorders of liver damage in the long run [23]. However, there are no known studies on copper exposure and neurodevelopment in children.

Heavy metal concentrations can be measured in many different matrices, some of which include blood, hair, urine, saliva, nails and fingerprints. Choosing the right matrix for heavy metal analysis depends on the investigation at hand. Hair and nail samples are less invasive, especially in child populations, and can be stored at room temperature until the chemical analysis [24]. Hair samples have been widely used in monitoring heavy metals and are a recognized biomarker, especially for mercury [25,26]. However, nails are also recognized as a matrix for exposure assessment [24]. The concentrations of elements in nails have been studied in relation to health problems such as skin diseases, hypertension, diabetes, obesity, diet, smoking and drinking habits [27,28,29,30,31,32]; however, very few in relation to behavior problems. The association of behavior problems in Pacific Island children and toenail mercury exposure has been explored [33]; however, other heavy metals such as Ni, Cu, Pb, Al, Cr and Cd and behavior problems need to be further understood within Pacific Island children. Therefore, the purpose of this sub-study was to investigate the association of toenail Ni, Cu, Pb, Al, Cr and Cd, along with other risk factors, with behavior problems in Pacific Island children at nine years of age.

## 2. Materials and Methods

A sub-sample of nine-year-old Pacific children within Pacific Island Families (PIF), a longitudinal birth cohort, were invited to participate in this sub-study. The details of the PIF cohort profile is further provided elsewhere [34]. All participants that were contacted provided assent/consent (n = 278) to be a part of this sub-study. The main PIF cohort was selected from South Auckland, which has the highest Pacific Island population in New Zealand and is representative of the Pacific Island Families living in NZ [34]. Children with short nails was an exclusion criterion; however, all included participants provided toenail samples. The NZ Health and Disability Ethics Committee (NTX/07/05/050) provided approval for this study.

The toenail samples (Ω > 50 milligram (mg)) were collected from the children at their school. Each participant’s nails were placed in a polythene bag, with their identification numbers (for anonymity) and kept at room temperature until chemical analysis was conducted. Toenail sample analysis has been explained further elsewhere [35]. However, all nails were washed prior to chemical analysis to remove any external dirt using acetone, deionized distilled water (DDW, 18.2 MΩ) (×3) and then acetone again. An analysis of all the washed and digested nail samples was carried out by inductively coupled plasma mass spectrometry (ICP MS) using an Agilent 7700 X ICP-MS instrument (Agilent Technology, Santa Clara, CA, USA). The recovery rate range for all the elements was between 76% and 101.3%. All instrumental data for each element (according to the isotope selected) was reported as counts per second. The value was corrected for a reagent blank signal (to correct for any contribution from the digestion procedure) and ratioed with the internal standard isotope value (to correct any instrumental drift or signal enhancement/depression caused by the matrix). Data for the calibration standards was handled in the same manner and an Excel™ calibration curve was produced for each element, with ratio signal (*y*-axis) and concentration of five standards (*x*-axis), from which the calibration equation was determined for calculation of the unknown toenail sample elemental concentration. The elemental values for each toenail sample were corrected for the dilution factor and the final values used in this data analysis. All sample were analyzed by using internationally recognized certified reference materials (NIST SRM 1643e (National Institute of Standards and Technology, Gaithersburg, MD, USA) and TMDA-54.4 (National Water Research Institute, Canada). Other data that was collected are addressed in the following subsections.

### 2.1. Questionnaires

The mother–child pairs who were part of this sub-study completed reliable and validated interviewer-administered questionnaires regarding: the sex of the child (male, female); the mother’s marital status (single, married, de facto); maternal question on prenatal smoking exposure (yes/no); the child’s Pacific ethnicity (Samoan, Tongan, Cook Island, and others including Tokelau and Niuean); family income (≤$20,000, $20,001–$40,000, >$40,000); the presence of any childhood health condition (yes/no).

### 2.2. Behavioral Problems

This parental version of the 120-item child behavior checklist (CBCL)/6-18 [36] was administered to the mothers or child carers as part for the main PIF cohort. The ratings were obtained based on emotional and behavioral problems in the children. The CBCL is a validated tool which is known for its cultural acceptance across many different cultures and countries [37]. The CBCL consists of internalizing behavior, externalizing behavior and total behavior which are determined by calculating the total problem scores (T-scores). The CBCL is assessed on a 3-point Likert-type scale: 0 = not true, 1 = somewhat or sometimes true, and 2 = very true or often true. Higher scores indicate greater degrees of behavioral and emotional problems. Using the cut-off criteria recommended by Achenbach and Rescorla [36], the 83rd percentile was used to define the borderline/normal group whilst the 90th percentile defined the clinical range group for the total scores. In all the behavioral categories, children were either in the normal group (scored below clinical range) or clinical group (scored within the clinical range). The children that fell within the clinical group were classified as having child behavior problems.

### 2.3. Anthropometric Measurement

The International Obesity Task Force (IOTF) criteria for obesity (normal, overweight, obese) was used in this study [38]. Prior to data collection, equipment was standardized, procedures documented in an operations manual and assessors trained.

### 2.4. Statistical Analysis

All data was imported into Microsoft Excel and transported to a statistical software SAS version 9.4 for statistical analysis. Each element of interest was reported as median, upper (75%) and lower (25%) quartiles and dispersion (range). Tests of the significance of bivariate associations between total behavior and other measures were derived using *t*-tests for independent samples or Mann–Whitney U-test, chi-square or Fisher’s exact tests of independence for comparisons of proportions. Univariate logistic regression was fitted for determining the risk factors associated with the behavior outcomes. Odds ratio estimates and corresponding confidence intervals were also derived using the fitted model parameters.

A probability *p* value of <0.05 was used to determine the level of statistical significance.

## 3. Results

### 3.1. Demographic Characteristics

Table 1 provides information on the characteristics of the study subjects. There was a sufficient mass of toenail clipping provided by the 278 participants for ICP-MS analysis. The study population included 160 boys (58%) and 118 (42%) girls with the majority being of Samoan ethnicity (53%). Almost a quarter of the children had a birth weight of above 4 kg. The majority of the children were either obese or overweight (67%). Almost a quarter of the children’s mothers smoked during pregnancy within this sample. The majority of the children did not have any childhood health conditions (82%). The proportion of subjects with behavioral problems in the clinical range (61/278 or 22%; 95% CI: 17% to 27%) was higher than expected given that “clinical range” was defined as having a CBCL total problem score above the population 90th percentile. No significant associations were found between any of the variables and behavioral problems.

### 3.2. Heavy Metal Concentrations

As reported in Table 2, no statistically significant associations were observed between heavy metal concentrations and behavior outcomes. However, Cu, Pb and Ni were higher (in both clinical and normal groups) than the laboratory reference value (LRV). Al and Cr concentrations were lower than the LRV in both the clinical and normal group.

For the total behavioral problems, based on the univariate results, only income was found to be significantly associated (Table 3). Therefore, for those earning between $20,000–$40,000 the odds of behavior problems was twice as much compared to those with ≤$20,000.

## 4. Discussion

The current study was conducted within the Pacific Island Families study birth cohort and used toenail samples as a matrix for determining the heavy metal burden within these children. The collection of toenail samples was a novel approach for this cohort. More research is required in order to understand the multi-elemental analysis using toenail clippings in epidemiological studies, where children are involved.

The majority of the children were males (58%) and more than half of the families in this study identified themselves as of Samoan origin (53%). It goes without saying that Pacific peoples are diverse, as seen in this cohort, and vary in their demographics, socio-cultural beliefs and practices [40]. Most of the participant children lived in homes with income levels below $40,000. The personal incomes of Pacific people are generally low compared to the rest of Auckland ($18,900 median personal income compared with $29,600) [40]. They are more likely to rent than own their own homes, with over 60% reporting problems with their rental houses [41]. Housing problems have been shown to be associated with psychological distress within the Pacific Island Family cohort [42]. According to the 2013 census, 36% of Pacific children were between the ages of 0 and 14 years [43] and were more likely to be living in lower decile areas than other ethnic groups, apart from Māori [44]. Living in lower decile areas and mental health disorders are correlated [45]. Poor housing conditions are common, especially in Auckland, NZ, where there is a housing crisis characterized by the undersupply of housing and increasing house prices [46]. With these house crises and the high percentage of Pacific people renting, the likelihood of water pipes leaching heavy metals or living close to traffic exposes them to toxic metals. As Pacific people in NZ play an important role socially and economically, reducing their burden of illness, such as mental health, becomes important. Mental health in children includes behavior issues, including internalizing or externalizing behaviors, which this study has explored.

- Behavioral problems and heavy metal levels

Cu is an essential element required by the body; however, in excess it can cause liver and kidney damage [47]. Cu exceeded the LRV [39] range within the study participants. People with Wilson’s disease are incapable of excreting Cu, leading to Cu toxicity [48]; however, none of the children in this cohort had Wilson’s disease. Exposure to Cu occurs generally through drinking water, especially if the pipes are made of Cu, or using Cu cookware. This was not measured within the cohort. There was no difference in the Cu concentrations between children with behavior problems and children without behavior problems. Some correlations have been observed between Cu and neurodevelopmental problems like schizophrenia [49], anxiety [50], and Parkinson’s disease [51]. However, these studies were conducted in the adult population and not children. Further research is required to study the long-term effects of high Cu levels and neurodevelopmental disorders in children.

Cr was not associated with total behavior problems in this study, as the concentrations were lower in both the clinical and normal group than the LRV [39]. Cr is generally found in animals, plants and dust particles and is considered a micronutrient [52]. Cr comes in many different forms; however, Cr III is an essential nutrient for humans in smaller amounts [53]. High Cr exposure in adults generally occurs through working in tanneries, as a machinist, or in printing [54]; however, the study participants would unlikely to be working in such environments. One recent cross-sectional study found an association between Cr and neuropsychological problems in children [55]. Further research is required to understand the effects on behavior, if any. Additionally, different forms of Cr have different health risks in humans; therefore, identifying each form of Cr and its effects on people is important.

Pb is a neurotoxin and is known to cause neurodevelopmental disorders even at low concentrations [56]. It is known that living closer to motorways, old paint around the houses, and dust particles expose people to heavy metals such as Pb. There is compelling evidence that for children an early adverse environment with Pb exposure can have lifelong effects on the emergence of conduct disorder, substance abuse, and physical and mental health problems [57]. Within this study, Pb levels were higher than the LRV [39], but we did not find any associations with behavior problems.

We did not find Al associated with behavioral problems in children. Al has been used for a long time for a number of different purposes due to its properties. Al is used in manufacturing processes, as crockery, and in jewelries, some medications and cosmetics [22]. Identification of use of crockery such as aluminum pots and pans was not determined for this study. A French longitudinal study demonstrated that Al exposure via drinking water showed a positive association with mild cognition over time [58]. Many other studies have shown an association of hair and urine aluminum levels and autism spectrum disorder as compared with control groups [59,60]. However, further research is required in this area in relation to neurodevelopment in children.

Cd can be found in animals and plants at minor levels [53] and is known to interfere by mimicking Zn and Ca, which are important in development [61]. Also, Cd exposure can occur via smoking or passive smoking [61]. Some studies have found an association between hair Cd levels and behavioral problems [62] as well as lower child intelligence [63] whilst some studies found no such associations with regards to hair Cd levels and behavior problems [64]. No associations were found on cord cadmium levels and children’s IQ at 4.5 years [65]. As there is mixed evidence on the effects of Cd on children’s neurodevelopment, further research is required. Within this study, no associations were observed between behavior problems and toenail Cd levels.

Some forms of Ni can be toxic to humans; however, there are not many studies that show adverse effects in humans. The Ni concentrations in the study participants were slightly higher than the LRV; however, we did not find any association with behavioral problems. Exposure to nickel can happen via food, soil, water and air [66]. Ni in soil was associated with neural-tube defects; however, other studies did not find any associations with prenatal Ni exposure and birth weight [67], nor was there any effect on genital malformation, musculoskeletal defects or birthweight [68]. A systematic review on Cr and Ni concluded that there is a weak evidence of Ni and neurodevelopmental outcomes such as autism [16].

- Demographic and lifestyle factors:

Maternal smoking in pregnancy was not associated with behavioral problems in the children within this study. In contrast, findings have been observed in other studies which have shown prenatal smoking elevates the risk of behavioral problems [69,70,71]. Some studies have reported social factors were responsible for behavior problems later in life, rather than just prenatal smoking exposure [72,73]. Most studies have focused only on prenatal smoking; however, more evidence is required to understand prenatal and postnatal exposure in children after taking into account societal factors. The apparent association (*p* = 0.028 without Bonferroni correction) of behavioral problems with moderate household income (but not with higher income) is counterintuitive. But this was the only finding of potential statistical significance among the 18 comparisons in Table 3, so it may be merely a chance association.

- Strengths and Limitations

One of the strengths of this study was the cultural competent nature of the research. The Pacific Island Families study is a cohort that uses Pacific people’s research approaches. For example, the engagement of a Pacific advisory group, the inclusion of the Pacific community in research activities, and the sharing the research outcomes within the community was an important part of the research process. It is important to recognize/engage with Pacific peoples and their cultures before undertaking research which builds equitable and meaningful research outcomes [74]. Additionally, more than just numerical values for the sample size were considered, including cultural beliefs and customs within the Pacific Island peoples [75]. The representation of Pacific ethnic groups in NZ was a strength in this study, as it was conducted within an internationally recognized Pacific Island cohort study. The use of toenail sample as a biomarker within this study was a strength as they are non-invasive and easy to store, although this might not be a good matrix for heavy metal and behavioral outcomes within this study. Other matrices need to be explored with regards to heavy metal exposure and behavioral problems. One of the limitations of this study is the cross-sectional study design, which limits establishing causality and temporality.

## 5. Conclusions

The results of this study show that there is a presence of heavy metal, particularly Cu, Ni and Pb. However, no association between behavioral problems and heavy metal was observed. Sociodemographic factors, specifically income levels, were associated with behavior problems in univariate analysis, but further research is required to ascertain this association. As health risks can increase due to increased heavy metal concentrations, regular environmental exposure monitoring should be conducted in order to lower the health risks.

## Figures and Tables

**Table 1:** Summary descriptive results for behavioral outcomes (normal: n = 217; clinical: n = 61) based on CBCL scores.

Variables		Normal n (%)	Clinical n (%)	Total	*p*-Value
Gender	Male	122 (56.22)	38 (62.3)	160	0.396
	Female	95 (43.78)	23 (37.7)	118	
Ethnicity	Samoan	110 (50.69)	38 (62.3)	148	0.138
	Tongan	24 (11.06)	5 (8.2)	29	
	Cook Island	40 (18.43)	13 (21.31)	53	
	Other	43 (19.82)	5 (8.2)	48	
Birthweight	<2500 g	6 (2.8)	0 (0)	6	0.171 *
	2500–2999	23 (10.75)	12 (20)	36	
	3000–3999	135 (63.08)	33 (55)	168	
	4000–5000	50 (23.36)	15 (25)	65	
Income	≤$20,000	73 (34.76)	20 (34.48)	93	0.172
	$20,001–$40,000	108 (51.43)	35 (60.34)	143	
	>$40,000	29 (13.81)	3 (5.17)	32	
Marital status	Single	59 (27.31)	17 (30.36)	76	0.853
	De facto	32 (14.81)	7 (12.5)	39	
	Married	125 (57.87)	32 (57.14)	157	
Smoking in pregnancy	Yes	51 (23.5)	18 (29.51)	69	0.337
	No	166 (76.5)	43 (70.49)	209	
Presence of any childhood health condition	No	174 (81.69)	52 (85.25)	226	0.519
	Yes	39 (18.31)	9 (14.75)	48	
Obesity **	Normal	75 (34.88)	15 (24.59)	90	0.138
	Overweight	57 (26.51)	14 (22.95)	71	
	Obese	83 (38.6)	32 (52.46)	115	

* Fisher’s exact test or chi-square test used; ** International Obesity Task Force (IOTF) obesity category.

**Table 2:** Median (interquartile range) of toenail heavy metal concentrations (µg/g) by child behavior checklist (CBCL) behavior outcomes.

Metals	Normal Median (Interquartile Range)	Clinical Median (Interquartile Range)	*p*-Value	Laboratory Reference Value ª (Mean)
Ni	0.33 (0.19, 0.78)	0.32 (0.18, 0.54)	0.535 *	0.2
Cu	17.03 (13.55, 22.66)	17.08 (13.1, 21.24)	0.516 *	<1.0
Pb	0.53 (0.12, 1.1)	0.47 (0.25, 0.92)	0.835 *	<0.05
Al	5.34 (3.87, 11.2)	5.63 (4, 11.2)	0.945 *	8.5
Cr	0.67 (0.46, 1)	0.76 (0.55, 1.15)	0.078 *	1.5
Cd	0.22 (0.13)	0.19 (0.11)	0.233	-

* Mann–Whitney test or two sample *t*-tests used; **ª** Ward (2008) [39].

**Table 3:** Univariate logistic regression result of total behavioral problem and associated covariate.

Covariate	Level	Odds Ratio (95% CI)	*p*-Value
Gender	Female	1.41 (0.84–2.37)	0.198
	Male	Reference	
Ethnicity	Tongan	0.51 (0.20–1.27)	0.053
	Cook Island	0.52 (0.26–1.05)	
	Other	0.42 (0.19–0.91)	
	Samoan	Reference	
Birthweight	3000–3999	0.82 (0.40–1.69)	0.766
	4000–5000	0.99 (0.43–2.25)	
	<2999	Reference	
Income	$20,001–$40,000	2.02 (1.12–3.62)	0.028 *
	>$40,000	0.90 (0.34–2.37)	
	≤$20,000	Reference	
Marital status	De facto	1.10 (0.46–2.61)	0.605
	Married	1.35 (0.73–2.48)	
	Single	Reference	
Smoking in pregnancy	Yes	0.65 (0.35–1.20)	0.171
	No	Reference	
Presence of any childhood health condition	Yes	1.39 (0.73–2.67)	0.316
	No	Reference	
Obesity **	Overweight Obese Normal	0.94 (0.41–2.15) 1.95 (0.99–3.88) Reference	0.075
Ni	Per unit increase	1.03 (0.97–1.10)	0.336
Cu	Per unit increase	1.01 (0.99–1.03)	0.518
Pb	Per unit increase	1.09 (0.90–1.31)	0.384
Al	Per unit increase	1.00 (0.96–1.04)	0.944
Cr	Per unit increase	1.09 (0.91–1.30)	0.363
Cd	Per unit increase	0.30 (0.04–2.46)	0.260

* Significant at the 5% level; ** IOTF obesity category.
